# Efficacy of a text messaging (SMS) based smoking cessation intervention for adolescents and young adults: Study protocol of a cluster randomised controlled trial

**DOI:** 10.1186/1471-2458-12-51

**Published:** 2012-01-19

**Authors:** Severin Haug, Christian Meyer, Andrea Dymalski, Sonia Lippke, Ulrich John

**Affiliations:** 1 Research Institute for Public Health and Addiction, Konradstrasse 32, 8031 Zurich, Switzerland; 2 University of Greifswald, Institute of Epidemiology and Social Medicine, Walther-Rathenau-Str. 48, 17487 Greifswald, Germany; 3 Jacobs Center on Lifelong Learning and Institutional Development, Jacobs University Bremen, Campus Ring 1, 28759 Bremen, Germany

## Abstract

**Background:**

Particularly in groups of adolescents with lower educational level the smoking prevalence is still high and constitutes a serious public health problem. There is limited evidence of effective smoking cessation interventions in this group. Individualised text messaging (SMS) based interventions are promising to support smoking cessation and could be provided to adolescents irrespective of their motivation to quit. The aim of the current paper is to outline the study protocol of a trial testing the efficacy of an SMS based intervention for smoking cessation in apprentices.

**Methods/Design:**

A two-arm cluster-randomised controlled trial will be conducted to test the efficacy of an SMS intervention for smoking cessation in adolescents and young adults compared to an assessment only control group. A total of 910 daily or occasional (≥ 4 cigarettes in the preceding month and ≥ 1 cigarette in the preceding week) smoking apprentices will be proactively recruited in vocational school classes and, using school class as a randomisation unit, randomly assigned to an intervention group (n = 455) receiving the SMS based intervention or an assessment only control group (n = 455). Individualised text messages taking into account demographic data and the individuals' smoking behaviours will be sent to the participants of the intervention group over a period of 3 months. Participants will receive two text messages promoting smoking cessation per week. Program participants who intend to quit smoking have the opportunity to use a more intensive SMS program to prepare for their quit day and to prevent a subsequent relapse. The primary outcome measure will be the proportion of participants with 7-day point prevalence smoking abstinence assessed at 6-months follow-up. The research assistants conducting the baseline and the follow-up assessments will be blinded regarding group assignment.

**Discussion:**

It is expected that the program offers an effective and inexpensive way to promote smoking cessation among adolescents and young adults including those with lower educational level and independent of their motivation to quit.

**Trial registration number:**

ISRCTN: ISRCTN19739792

## Background

Tobacco use is a major cause of disease burden and the single most preventable cause of death in the world [1,2]. Although smoking prevalence rates among adolescents of most European countries have declined within the last few years, smoking continues to be a serious problem, particularly in adolescents and young adults with lower educational level [3,4].

There is limited evidence of smoking cessation interventions demonstrating efficacy in adolescents and young adults [5,6]. In a Cochrane review of smoking cessation interventions for smokers who are younger than 20 years, approaches based on the *Transtheoretical Model *[7] achieved moderate long-term success, whereas the efficacy of psychosocial and pharmacological interventions has not been demonstrated so far [5]. A recent review [6] suggested delivering (1) smoking cessation programs for youth in contexts which are geared to youth such as schools or sports clubs, (2) interventions addressing cognitive-behavioural, motivational and social influence contents, and (3) programs with at least five sessions.

Electronic communication technology such as mobile phone text messaging (SMS) has the potential to deliver smoking cessation support to large population groups. In 2010, 98% of 12-19-year-old adolescents from Switzerland owned a mobile phone; use of the mobile phone was the most frequent leisure time activity in this population group. Furthermore, reading and sending of text messages were the most frequent activities when using the mobile phone [8].

By use of expert system technology that provides information based on individual demographic or smoking-related characteristics, electronic communication technology can be a viable, time- and cost-saving alternative to interpersonal counselling [9,10]. Particularly, SMS provides opportunities for individualised and interactive information delivery that may be accessed easily, independent of time and place.

To date, two randomised controlled studies were conducted to test the efficacy of SMS based smoking cessation interventions in adults motivated to quit smoking [11,12]. Within those studies, conducted in Great Britain and New Zealand, smokers who intended to quit within the subsequent month received motivational messages and behavioural-change support over a period of 26 weeks. The messages were matched to participants' demographic and smoking-related characteristics gathered at baseline. Additionally, participants could request instant messages aimed at craving or lapse situations. Five text messages per day were sent to the participants one week before and over four weeks after a predefined quit date that was negotiated with each participant. After this period, the intervention became less intensive, with the number of messages sent being reduced from five a day to three a week until the end of the 26th week. While intention-to-treat analyses could not reveal a significant intervention effect at the 6-months follow-up in the study by Rodgers et al. [12], Free et al. [13] found significantly higher abstinence rates in the intervention than in the control group (9% vs. 4%).

Within two pilot studies in which young adult smokers, irrespective of their motivation to quit, were proactively invited to an SMS based smoking cessation intervention, high participation and retention rates could be achieved [14,15]. To date, no randomised controlled trials testing the efficacy of smoking cessation interventions employing SMS in adolescents and young adults, or trials testing the efficacy of SMS interventions in smokers irrespective of their motivation to quit, have been reported.

Our aim is to outline the study protocol of a trial testing the efficacy of an SMS based intervention for smoking cessation in apprentices, irrespective of their motivation to quit. Apprentices have been chosen as the target population since evidence revealed high smoking prevalence rates in this subgroup of adolescents and young adults with heterogeneous educational level [4,16].

## Methods/Design

### Design and hypotheses

**Figure 1:**
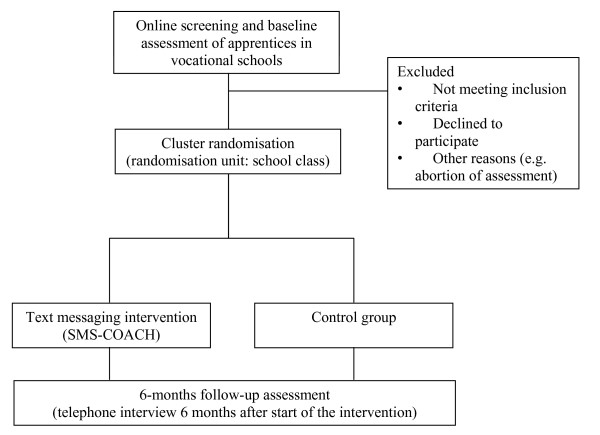
**Study design**.

A two-arm cluster-randomised controlled trial will be conducted to test the efficacy of a text messaging based intervention for smoking cessation in adolescents and young adults compared to an assessment only control group. Apprentices within vocational schools will be proactively invited for study participation irrespective of their intention to quit. The smoking cessation text messages will be based on the *Health Action Process Approach *(HAPA, [17]) considering cognitive-behavioural and motivational components. Text messages will be sent to the participants over a period of three months and will be tailored according to data gathered at baseline and a weekly SMS assessment. At the six months follow-up, we expect a higher 7-day point prevalence smoking abstinence rate in apprentices of the intervention group than in those of the assessment only control group. Secondary outcome measures will be 30-day point prevalence smoking abstinence rate, self-efficacy for smoking cessation, nicotine dependence, and quit attempts. An overview of the study design is presented in Figure 1.

### Participant recruitment and baseline assessment

Smoking apprentices will be recruited at vocational schools in Switzerland. Contact teachers for addiction prevention in approximately 30 Swiss vocational schools will be invited to participate with some of their classes in a study testing the efficacy of a text messaging based smoking cessation program. Participating contact teachers will schedule one school hour per school class for the screening of eligibility criteria, study information, and baseline assessment. Study participants will be recruited by study assistants (graduate students of psychology). The study assistants will invite all apprentices from a school class to participate in an online health survey during a regular school lesson, reserved for health education. Furthermore, they will inform the apprentices that some persons will be invited to participate in a study testing the efficacy of a text messaging intervention for health promotion. To decrease reporting bias the study assistants will not provide more information about the purpose of the study before the screening of eligibility criteria will be completed. Afterwards the apprentices will be invited to fill in an online screening. The screening includes the assessment of demographic data, alcohol consumption, weekly physical activity, smoking status, and ownership of a mobile phone. Inclusion criteria for study participation are (1) daily or occasional cigarette smoking (at least 4 cigarettes in the preceding month and at least one cigarette during the preceding week) and (2) ownership of a mobile phone. Subsequently, eligible persons will be informed about the aim of the study, the intervention arms, assessments, reimbursement, and data protection. Study information will be provided online and in paper form by the study assistants. Reimbursement of 10 Swiss francs (~ € 8.00) for participation at the follow-up assessment after 6 months will be offered to all study participants. Reimbursement of 1 Swiss franc (~ € 0.80) will be offered to the participants of the intervention group for each SMS response to the weekly SMS assessments within the program. After receiving informed consent online, all study participants will be invited to choose a username and to provide their mobile phone number. Furthermore, the following smoking related variables will be assessed: intention to quit, daily/weekly cigarette consumption, time to first cigarette in the morning, past quit attempts, smoking status of friends and parents, and age of smoking onset.

### Ethical review

The study protocol was approved by the Local Ethics Committee of the Canton of Zurich, Switzerland. The trial will be executed in compliance with the Helsinki Declaration.

### Randomisation and allocation concealment

To avoid spill-over effects within school classes, we will conduct a cluster-randomised controlled trial using school class as a randomisation unit. Due to the heterogeneity of apprentices in the different vocational schools (e.g. concerning gender or professions), we will use separate randomisation lists for each vocational school (stratified randomisation). Furthermore, to approximate equality of sample sizes in the study groups, we will use block randomisation with computer generated randomly permuted blocks of 4 cases [18].

The study assistants supervising the baseline assessment in the vocational schools will be blinded concerning group allocation of the school classes. Also, group allocation will not be released to study participants until having provided informed consent, their username, mobile phone number, and baseline data of the above mentioned smoking-related variables. Furthermore, the study assistants conducting the computer assisted telephone interviews at follow-up will be blinded when assessing the primary and secondary outcome measures.

### Sample size calculation

Based on results of a similar study which tested the efficacy of proactive telephone counselling for smoking cessation in high school students [19], we expect an 8% difference in 7-day point prevalence abstinence rates between the intervention and the control condition at 6-months follow up assessment (25% versus 17%, respectively). A sample size of n = 406 in each study group would have 80% power for a Chi-Square Test (*α *= 5%, 2-sided) in order to detect this difference based on a calculation using *G-Power*. As apprentices are nested within school classes, we additionally needed to consider a potential design effect of 1.12 (assumed class size: 20 apprentices; 35% study participants resulting in an average cluster size of n = 7; intra-cluster correlation coefficient: 0.02) resulting in a required sample size of n = 455 per study group and a total of N = 910 study participants.

### Intervention

#### Technological background

The intervention program named SMS-COACH is fully automated and based on Internet technology using a LAMP system (Linux, Apache, MySQL, and PHP). The program used in the present study is an extended and modified version of a previous text messaging program for smoking cessation which was successfully tested in 2 pilot studies [14,15]. All incoming and outgoing text messages will be automatically recorded. Incoming messages will be analysed immediately 24 hr/day.

#### Theoretical background

Primarily, the program is based on the HAPA [17]. This health behaviour model suggests a distinction between motivation processes resulting in goal setting (people in this stage are called preintenders) and volition processes leading to the actual health behaviour. The latter is further subdivided into those people being inactive (intenders) and those who already adopted the health behaviour (actors). Within the initial preintentional stage, outcome expectancies (pros of stopping smoking and cons of further smoking), risk perception, and perceived self-efficacy are seen as important social-cognitive predictors in order to develop an intention to act. Within the subsequent intentional stage, planning processes are crucial in order to achieve the desired action. Once an action has been initiated, self-regulatory skills are important, e.g. how to cope with craving situations, in order to maintain the health behaviour. Beyond the HAPA we integrated intervention elements derived from the *Social Norms Approach *[20] as well as implementation intentions, which are if-then plans that link situational cues with responses that are effective in attaining a desired outcome [21].

#### Intervention elements

The intervention program consists of (1) an online assessment of the individual smoking behaviour and smoking related attitudes, (2) a weekly SMS-assessment of smoking-related target behaviours, (3) two weekly text messages tailored to the data of the online and the SMS-assessments, and (4) an integrated quit day preparation and relapse prevention program.

#### Online assessment

Beyond the screening questions and the assessment of the above mentioned smoking related variables, which will be assessed in both study groups, participants of the intervention group will additionally receive online questions assessing (1) outcome expectancies of smoking cessation, (2) situations in which craving for cigarettes occur, (3) alternative strategies to handle these craving situations, and (4) costs per pack of cigarettes.

#### Weekly SMS assessment

During the 3-month intervention period, participants of the intervention group will receive one text message per week for the assessment of smoking related target behaviour. This question can easily be answered by typing a single letter or number, and using the reply function of the mobile phone. The weekly SMS assessment will be sent at a fixed point in time each week (6 p.m. at the weekday of registration for the program). The content of the question will depend on the HAPA stage as well as on the number of the intervention week.

For all participants, the HAPA stage will be assessed in even weeks by the question: "Have you recently smoked cigarettes?" with the following response options (1) "Yes, and I do not intend to quit" (preintentional stage), (2) "Yes, but I am considering to quit" (preintentional stage), (3) "Yes, but I seriously intend to quit" (intentional stage), or (4) "No, I quit smoking" (action stage). This question assesses both smoking status and intention to quit over time and the responses to this question allow tailoring the SMS feedbacks according to the current HAPA stage [22].

In odd weeks, we will assess the number of cigarettes smoked per day or week (depending on smoking status: daily/occasionally) in smokers of the preintentional stage; and, we will assess whether smokers in the intention or action stage applied the individually chosen strategies to cope with craving situations (e.g. "Did you apply the following strategy recently? When I am on a party, I distract myself from smoking by dancing.").

#### Individually tailored text messages

On the first level, the text messages are tailored to the HAPA stage (preintentional, intentional, action), defined by current smoking status and intention to quit smoking. Persons in the preintentional stage will receive text messages addressing (1) risks of smoking, (2) monetary costs of smoking, (3) social norms of smoking, (4) outcome expectancies, and (5) motivation to reduce the number of cigarettes smoked per day (daily smokers) or week (occasional smokers). Persons in the intentional stage will receive text messages which (1) motivate to use social support for smoking cessation, (2) provide strategies to cope with craving situations, and (3) provide tips for preparing smoking cessation (e.g., reducing the number of cigarettes, identifying craving situations). Persons in the action phase will receive text messages (1) motivating to reward themselves for staying abstinent, (2) providing strategies to cope with craving situations, and (3) motivating to use social support for staying abstinent.

On the second level, the text messages are tailored according to the individual information provided at the baseline assessment as well as through the weekly SMS assessments. Exemplary text messages are displayed in Table 1.

**Table 1:** Exemplary text messages

HAPA stage	Content category	Exemplary text message
Preintentional Stage	Social Norms	Hey Mika. Did you know, not smoking is in! A survey from the University of Zurich found that among 16-to-17-year old female adolescents, only 11% still smoke cigarettes daily.
	
	Outcome Expectancies	Hello Mika. You have the opinion, that after having stopped smoking you will be able to breathe deeply more easily again. That's totally correct! Already after the first weeks you are going to notice strong changes: you will be able to breathe more freely, will be fitter at sports and you will be less susceptible to diseases.
	
	Monetary costs of smoking	You smoke approximately 8 cigarettes less in comparison to the beginning of the program SMS-COACH. Thus, you save approximately 70 Swiss francs per month.

Intentional Stage	Social support for smoking cessation	There are forums online in which smokers and ex-smokers exchange their experiences on smoking cessation. Have a look at www.stop-tabac.ch
	
	Preparing smoking cessation	Hi Peter. Maybe you can avoid smoking cigarettes in situations in which you usually smoke by keeping yourself busy. For example, when having a break or waiting for the bus it can be extremely helpful to have a chewing gum or write an SMS.

Action Stage	Reward for staying abstinent	You can be proud of yourself to not smoke anymore. Reward yourself by buying something that you have desired for a long time.

	Coping with craving situations	Great job, Vince: journals or a book can help you to bridge time when waiting. If there is a moment in which you have nothing readily to hand, you can also distract yourself by using your cell phone for calling somebody or sending a text message to someone.

#### Integrated program for quit day preparation and relapse prevention

Persons in the intentional and action stage will have the possibility to additionally participate in an integrated program for quit day preparation and relapse prevention. Program participants in these phases will be informed biweekly about this option. After entering a scheduled quit day (e.g. "08.12.2011") this program will provide up to two daily text messages (weeks -1 to +1: two daily SMS; weeks +2 and +3: one daily text message) in order to prepare for the quit day and to prevent a subsequent relapse. For example, at the evening prior to the scheduled quit day, participants will receive the following text message: "SMS-COACH EXTRA: Good evening Jona. Tomorrow is your first smoke-free day. Throw away all cigarettes, ashtrays, and lighters tonight. Buy some peppermint sweets or chewing gums for tomorrow."

Study participants of the assessment only control group will not receive any of the above described intervention elements of the SMS-COACH.

### Outcomes

Computer assisted telephone interviews will be conducted at the six months follow-up assessment by trained interviewers. The primary outcome measure will be *7-day point prevalence smoking abstinence*, i.e. not having smoked a puff within the past seven days preceding the follow-up [23].

Secondary outcome measures will include *30-day point prevalence smoking abstinence, self-efficacy for smoking cessation *(action self-efficacy according to the HAPA-model [17]), *nicotine dependence *assessed by the *Heaviness of Smoking Index *(using self-reported time to the first cigarette of the day and number of cigarettes smoked per day [24]), and *quit attempts *within the past six months preceding the follow-up.

### Data analyses

We will use regression models to test the efficacy of the intervention on the different outcome measures. If necessary, we will control for baseline differences by adding additional baseline variables as covariates to the regression models. All analyses will be performed considering the intention-to-treat principle. Given the clustered nature of the data (apprentices within school classes) we will compute robust variance estimators for all regression analyses.

## Discussion

This study protocol presents the design of a cluster randomised controlled trial testing the efficacy of a text messaging intervention to increase smoking abstinence among adolescent or young adult current smokers. This is the first controlled trial testing the efficacy of a text messaging based smoking cessation intervention in a population consisting mainly of adolescents. Due to the realization of proactive participant recruitment, it is also the first trial testing the efficacy of a text messaging based smoking cessation intervention in smokers irrespective of their motivation to quit.

A limitation of the study is that the smoking status will be assessed by self-report and will not be biochemically verified. However, it is expected that a potential over-reporting of smoking abstinence will be independent of the study condition. According to the recommendations of the *Society for Research on Nicotine and Tobacco *(SRNT) subcommittee on biochemical verification of tobacco use and cessation [25] there are circumstances under which the added precision gained by biological validation is offset in such a way that its use is not required and may not be desirable. Examples given by the SRNT are population-based studies with low demand on smokers to quit, e.g. interventions with limited face-to-face contact and studies where the optimal data collection methods are through mail, telephone or Internet.

Given that the text messaging intervention is effective, it would provide an attractive, cost-effective model to increase smoking abstinence rates among adolescents and young adults including those with lower educational level and independent of their motivation to quit. Both the baseline assessment and the program registration are possible from every computer with Internet access and take only approximately ten minutes. Therefore, the monetary costs for the provision of the intervention are very low. The program could be easily disseminated across schools nationwide or via national prevention campaigns.

## Competing interests

The authors declare that they have no competing interests.

## Authors' contributions

SH, CM, and UJ were responsible for the study design. SH, SL, CM, AD and UJ developed the text messaging program SMS-COACH. SH and AD are responsible for the data collection. All authors read and approved the final manuscript.

## Pre-publication history

The pre-publication history for this paper can be accessed here:

http://www.biomedcentral.com/1471-2458/12/51/prepub
